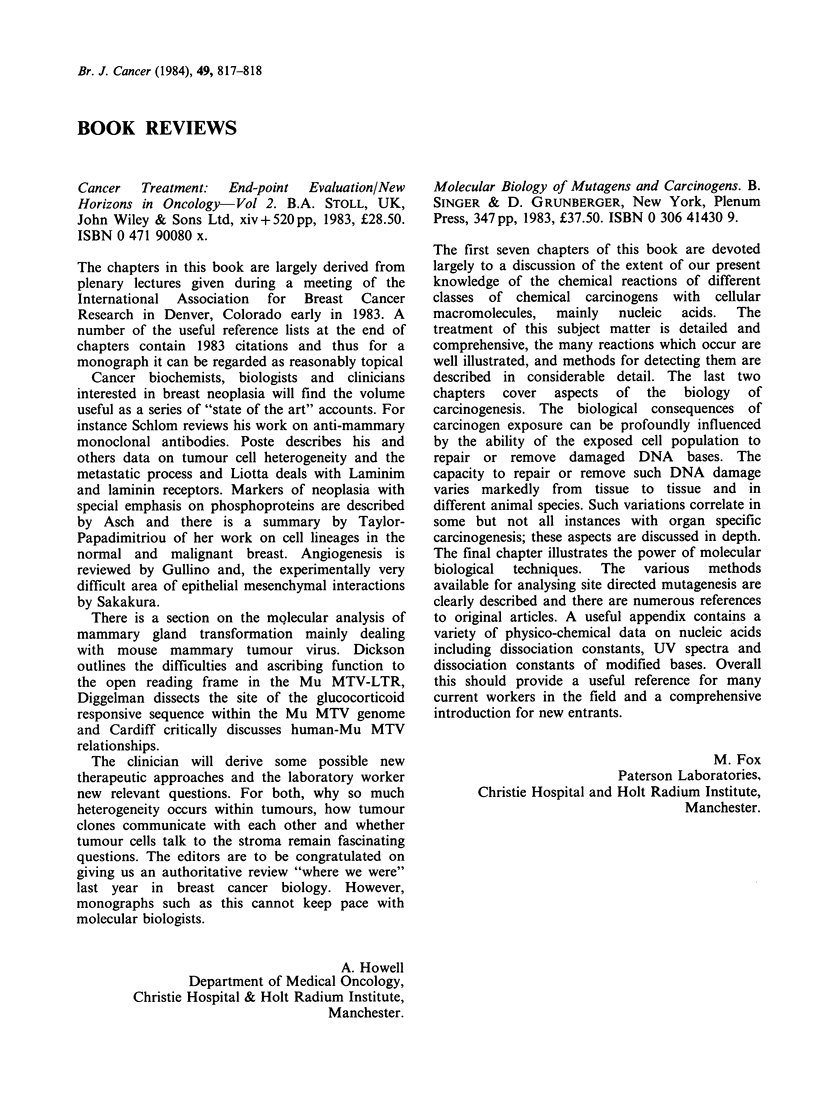# Cancer Treatment: End-point Evaluation/New Horizons in Oncology

**Published:** 1984-06

**Authors:** A. Howell


*Br. J. Cancer* (1984), **49**, 817–818

### BOOK REVIEWS

*Cancer Treatment: End-point Evaluation/New Horizons in Oncology—Vol 2.* B.A. Stoll, UK, John Wiley & Sons Ltd, xiv+520 pp, 1983, £28.50. ISBN 0 471 90080 x.

The chapters in this book are largely derived from plenary lectures given during a meeting of the International Association for Breast Cancer Research in Denver, Colorado early in 1983. A number of the useful reference lists at the end of chapters contain 1983 citations and thus for a monograph it can be regarded as reasonably topical

Cancer biochemists, biologists and clinicians interested in breast neoplasia will find the volume useful as a series of "state of the art" accounts. For instance Schlom reviews his work on anti-mammary monoclonal antibodies. Poste describes his and others data on tumour cell heterogeneity and the metastatic process and Liotta deals with Laminim and laminin receptors. Markers of neoplasia with special emphasis on phosphoproteins are described by Asch and there is a summary by Taylor-Papadimitriou of her work on cell lineages in the normal and malignant breast. Angiogenesis is reviewed by Gullino and, the experimentally very difficult area of epithelial mesenchymal interactions by Sakakura.

There is a section on the molecular analysis of mammary gland transformation mainly dealing with mouse mammary tumour virus. Dickson outlines the difficulties and ascribing function to the open reading frame in the Mu MTV-LTR, Diggelman dissects the site of the glucocorticoid responsive sequence within the Mu MTV genome and Cardiff critically discusses human-Mu MTV relationships.

The clinician will derive some possible new therapeutic approaches and the laboratory worker new relevant questions. For both, why so much heterogeneity occurs within tumours, how tumour clones communicate with each other and whether tumour cells talk to the stroma remain fascinating questions. The editors are to be congratulated on giving us an authoritative review "where we were" last year in breast cancer biology. However, monographs such as this cannot keep pace with molecular biologists.

A. Howell
Department of Medical Oncology,
Christie Hospital & Holt Radium Institute,
Manchester.